# 

**Published:** 1859-11

**Authors:** 


*New Publications Received.*—A Practical Treatise on the Diagnosis, Pathology, and Treatment of Diseases of the Heart. By Austin Flint, M. D., Professor of Clinical Medicine, etc., in the New Orleans School of Medicine ; Visiting Physician to the New Orleans Charity Hospital ; Honorary Member of the Medical Society of Virginia, of the Kentucky State Medical Society, of the Medical Society of Rhode Island, of the Pathological Society of Philadelphia, etc. Philadelphia : Blanchard & Lea. 1859.

A System of Surgery ; Pathological, Diagnostic, Therapeutic, and Operative. By Samuel D. Gross, M. D., Professor of Surgery in the Jefferson Medical College of Philadelphia ; Member of the American Philosophical Society ; Fellow of the College of Physicians of Philadelphia ; Corresponding Member of the New York Academy of Medicine, and of the Imperial Royal Medical Society of Vienna ; Author of a Treatise on the Urinary Organs, etc., etc., etc. Illustrated by nine hundred and thirty-six engravings, in two volumes. Philadelphia : Blanchard and Lea. 1859. From the publishers.

An Introduction to Practical Pharmacy ; designed as a Text Book for the Student, and as a Guide for the Physician and Pharmaceutist. With many Formulas and Prescriptions. By Edward Parrish, Graduate in Pharmacy, Member of the Philadelphia College of Pharmacy, and of the American Pharmaceutical Association ; and Principal of the School of Practical Pharmacy, Philadelphia. Second edition, greatly enlarged and improved. With two hundred and forty-six illustrations. Philadelphia : Blanchard & Lea. 1859. From the publishers.

Pathological and Practical Observations on Diseases of the Alimentary Canal, Œsophagus, Stomach, Cæcum and Intestines. By S. O. Habershin, M. D., London ; Fellow of the Royal College of Physicians ; Assistant Physician to Guy's Hospital, and Lecturer on Materia Medica and

Therapeutics; late Demonstrator of Morbid Anatomy, and Curator of the Museum at Guy's, etc., etc. Philadelphia: Blanchard & Lea. 1859. From the publishers.

The Action of Medicines on the System; or, the Mode in which Therapeutic Agents introduced into the Stomach produce their peculiar Effects on the Animal Economy. Being the Prize Essay to which the Medical Society of London awarded the Fothergillian Gold Medal for 1852. By Frederic William Headland, M. D., B. A., F. L. S.; Licentiate of the Royal College of Physicians, etc., etc. Third edition, revised and enlarged. Philadelphia: Lindsay & Blakiston. 1859. From the publishers.

Alcohol: Its Place and Power. By James Miller, Professor of Surgery in the University of Edinburgh, Surgeon in Ordinary to the Queen for Scotland, etc., etc. From the nineteenth Edinburgh edition. Philadelphia: Lindsay & Blakiston. 1859. From the publishers.

The Physician's Hand-Book of Practice for 1860. By William Elmer, M. D., and Louis Elsberg, M. D. New York: W. A. Townsend & Co., No. 46 Walker street. 1860. From the publishers.

The above publication will receive early notice.

☞ An advertisement, inconsistent with the character of the Journal, was inadvertently bound in with our last issue. This was not noticed until some days after the Journal had been sent out. Such an accident will never occur again.

☞ Prof. Austin Flint has now left New York to enter upon the duties of his chair in the New Orleans School of Medicine. He expects to return early in the spring, and until then, correspondents will please address him at New Orleans.

Our office and residence is still at 111 East 21st street, Gramercy Park House.

